# Intractable diseases treated with intra-bone marrow-bone marrow transplantation

**DOI:** 10.3389/fcell.2014.00048

**Published:** 2014-09-02

**Authors:** Ming Li, Kuquan Guo, Susumu Ikehara

**Affiliations:** ^1^Department of Stem Cell Disorders, Kansai Medical UniversityHirakata City, Japan; ^2^Department of Cardiac Surgery, Beijing Institute of Heart, Lung and Blood Vessel Disease, Beijing Anzhen Hospital Affiliated to Capital Medical UniversityBeijing, China

**Keywords:** intra-bone marrow-bone marrow transplantation, mesenchymal stem cell, rheumatoid arthritis, malignant tumors, autoimmune diseases

## Abstract

Bone marrow transplantation (BMT) is used to treat hematological disorders, autoimmune diseases (ADs) and lymphoid cancers. Intra bone marrow-BMT (IBM-BMT) has been proven to be a powerful strategy for allogeneic BMT due to the rapid hematopoietic recovery and the complete restoration of T cell functions. IBM-BMT not only replaces hematopoietic stem cells (HSCs) but also mesenchymal stromal cells (MSCs). MSCs are multi-potent stem cells that can be isolated from bone marrow (BM), umbilical cord blood (UCB), and adipose tissue. MSCs play an important role in the support of hematopoiesis, and modify and influence the innate and adaptive immune systems. MSCs also differentiate into mesodermal, endodermal and ectodermal lineage cells to repair tissues. This review aims to summarize the functions of BM-derived-MSCs, and the treatment of intractable diseases such as rheumatoid arthritis (RA) and malignant tumors with IBM-BMT.

## Introduction

Mesenchymal stromal cells (MSCs) are multi-potent progenitor cells mainly isolated from bone marrow (BM) (Campagnoli et al., 2001), adipose tissue (Zuk et al., 2001), and the umbilical cord (UC) (Erices et al., 2000). MSCs have been shown to differentiate into osteoblasts, adipocytes (Dominici et al., 2006), cardiomyocytes (Makino et al., 1999), and pancreatic islets (Tang et al., 2004). Furthermore, MSCs have the ability to migrate to injured tissue of liver (van Poll et al., 2008) and heart (Yokokawa et al., 2008). Adipose tissue and BM are the most readily available sources of MSCs because they are easy to harvest, and there are no ethical concerns. BM-derived MSCs (BMMSCs) have a higher degree of commitment to differentiate into chondrogenic and osteogenic lineages than adipose tissue-derived MSCs (Gimble et al., 2007), although there appears to be no difference between adipose tissue-derived MSCs and BM MSCs in terms of immunoregulatory functions and support of hematopoiesis (Poloni et al., 2012). On the other hand, BM MSCs modulate the immune response, suppress allogeneic T cell responses, and prevent the development of graft-versus-host disease (GVHD) (English, 2013).

BM transplantation (BMT) is useful for treating hematopoietic disorders, allogeneic BMT also being used to treat autoimmune diseases (ADs) (Nishimura et al., 1994). Intra-bone marrow-bone marrow transplantation (IBM-BMT) has been proven to be the most effective approach to treating allogeneic BMT, since IBM-BMT can replace not only hematopoietic stem cells (HSCs) but also BMMSCs Thus hematopoietic recovery is rapid, and no GVHD develops even if whole BM cells are injected (Kushida et al., 2001; Ikehara, 2003). In this review, we focus on rheumatoid arthritis (RA) and malignant tumors treated with IBM-BMT.

## Immunoregulatory functions of BMMSCs

BMMSCs have been reported to have the ability to modify and influence almost all the cells of the innate and adaptive immune systems mediated by BMMSC soluble factors, including IL-6, M-CSF, IL-10, TGFβ, HGF, and PGE2 (Aggarwal and Pittenger, 2005; Beyth et al., 2005; Ramasamy et al., 2007). The adaptive immune system, which is composed of T and B lymphocytes, generates specific immune responses to pathogens with the production of memory cells. BMMSCs modulate the function of dendritic cells (DCs), indirectly regulate T and B cell activity, and delay or prevent the development of acute GVHD (Zhang et al., 2009). BMMSCs have also been shown to suppress the differentiation of DCs and their function during allogeneic islet transplantation (Urban et al., 2008; Aldinucci et al., 2010). BMMSCs strongly inhibited the maturation and functioning of monocyte-derived DCs by interfering selectively with the generation of immature cells via inhibitory mediator of MSC-derived PGE2 (Lee et al., 2006). PGE2 has been identified as one of the candidates responsible for T cell inhibition by BMMSCs, and may have an immunostimulatory role by facilitating Th1 differentiation and expanding the Th17 T cell population (English et al., 2009; Yao et al., 2009). The expression of PGE2 was shown to be upregulated by IFNγ and TNFα in the BMMSCs for immunomodulatory function (English et al., 2007). BMMSCs can inhibit the cytotoxic effects of antigen-primed cytotoxic T cells by suppressing the proliferation and activity (Zhao et al., 2005) via the inhibition of the nuclear translocation of nuclear factor-kappa B (Matsuda-Hashii et al., 2004). BMMSCs have been shown to alter the NK cell phenotype and suppress proliferation of NK cells via the secretion of TGFβ 1 and PGE2, and via cytotoxicity against HLA class I-expressing targets (Aggarwal and Pittenger, 2005; Sotiropoulou et al., 2006; Ryan et al., 2007; Uccelli et al., 2008). BMMSCs have also been shown to inhibit the proliferation of B cells when stimulated with anti-CD40L and IL-4 (Glennie et al., 2005). One report has suggested that allogeneic BMMSCs inhibit the activation, proliferation and IgG secretion of B cells in a BXSB mouse model of human systemic lupus erythematosus (Deng et al., 2005).

Allogeneic BMMSCs are effective in the treatment of murine models of human disease (Zappia et al., 2005; Ding et al., 2009; Fiorina et al., 2009). BMMSCs were shown to be able to secrete regulatory cytokines that affect regulatory T cells, and to modulate the immunological dysregulation observed in antibody producing B cells and cytotoxic NK cells in the NOD mouse (Anderson and Bluestone, 2005). BMMSCs promote the endogenous repair of pancreatic islets and renal glomeruli in a streptozotocin-induced diabetic mouse model (Lee et al., 2006). Co-infusion of BMMSCs and BM cells was shown to inhibit the beta cell-specific T cell proliferation and to restore insulin and glucose levels (Urban et al., 2008). BMMSCs secrete many cytokines and growth factors such as HGF, which shows anti-apoptotic activity in hepatocytes and plays an essential part in the regeneration of the liver (Trim et al., 2000; Matsuda-Hashii et al., 2004). BMMSCs have also been shown to protect against experimental liver fibrosis in CCl4-induced rats (Zhao et al., 2005), and to suppress CD3 T-cell proliferation in collagen-induced arthritis (Schurgers et al., 2010).

In mammals, there are seven sirtuin family members, named Sirt1-7. Sirtuins plays a critical role in the regulation of fundamental biological responses to nutritional and environmental stimuli in each subcellular compartment (Blander and Guarente, 2004; Imai and Guarente, 2010). Sirt1 is a class III protein deacetylase, and Sirt1 activity can be regulated through NAD^+^. Sirt1 binds to and deacetylates a number of important transcription factors—such as peroxisome proliferator-activated receptor gamma (PPARγ), PPARα, PPAR gamma coactivator 1 alpha (PGC-1α), and the forkhead box, subgroup O (FOXO) family of transcription factors—to drive metabolic responses such as insulin secretion, gluconeogenesis, and fatty acid oxidation (Haigis and Sinclair, 2010). Some reports indicate that Sirt1 promotes osteogenesis and decreases adipogenesis of BMMSCs *in vitro* (Tseng et al., 2011; Peltz et al., 2012; Puri et al., 2012).

Sirt1 deacetylates β-catenin to regulate differentiation of MSCs in MSCs specific Sirt1 knock-out mice (MSC KO) (Simic et al., 2013). Moreover, Sirt1 has been shown to directly downregulate *Sost* gene expression, and promote bone formation in the treatment of osteoporosis (Cohen-Kfir et al., 2011). One report has shown that CD8 T cell differentiation is regulated by basic leucine zipper transcription factor, ATF-like (BATF), which is a member of the AP-1 family, via Sirt1 expression, BATF deficiency inducing high levels of Sirt1 expression in memory CD8 T cells but not in naive CD8 T cells (Kuroda et al., 2011).

## IBM-BMT

We reported that MRL/lpr mice possess abnormal radioresistant stem cells and have provided impressive evidence regarding the origin of ADs in this strain (Ikehara et al., 1989). BMT plus bone graft, which can recruit donor stroma cells, can prevent the recurrence of ADs (Ishida et al., 1994). However, allogeneic BMT + bone grafts failed to treat ADs in MRL/lpr mice, because these mice become more radiosensitive after the onset of lupus nephritis. Moreover, our previous reports showed that stroma cells can be trapped in the liver when BM cells are injected via the portal vein. Thus, directly injecting whole BM cells into the BM, as in IBM-BMT, has been shown to be a powerful strategy for the treatment of ADs in MRL/lpr mice. IBM-BMT, which not only replaces HSCs but also MSCs, has been proven to be the best method for allogeneic BMT: (1) hematopoietic recovery is rapid because the MSCs directly home to the bone cavity, (2) the restoration of T cell functions is complete even in donor-recipient combinations across the MHC barriers, and (3) no graft failure occurs even if the radiation dose is reduced (Kushida et al., 2001). Moreover, IBM-BMT of young marrow cells reversed the reduction of pro-B cells and pre-B cells. The frequency of follicular-B cells in the IBM-BMT group was significantly increased compared to the old group (Hida et al., 2010). We have already used IBM-BMT to successfully treat ADs, osteoporosis, diabetes, Alzheimer's disease, and for the induction of tolerance for organ transplantation (Takada et al., 2006; Guo et al., 2008; Kushida et al., 2009; Li et al., 2009, 2010) (Table 1).

**Table 1:** **IBM-BMT treatment of various diseases and induction of tolerance for organ transplantation**.

**Authors**	**Animal model**	**Effect of IBM-BMT**
Li et al., 2012	Mouse	Improve renal function
Zhang et al., 2012	Mouse	Prevention of leukemia
Shi et al., 2011	Mouse	Diminish risk of GVHD
Feng et al., 2010	Mouse	Prevention of premature ovarian failure
Li et al., 2009	Mouse	Amelioration of cognitive ability
Kushida et al., 2009	Mouse	Prevention of rheumatoid arthritis
Okazaki et al., 2008	Mouse	Liver transplantation
Miyake et al., 2008	Mouse	Prevention of GVHD
Abraham et al., 2008	Mouse	Prevention of type 2 diabetes
Guo et al., 2008	Rat	Long-term donor specific tolerance in cardiac allograft
Feng et al., 2007	Mouse	Prevention of osteoporosis and hypogonadism
Koike et al., 2007	Mouse	Suppression of growth of colon cancer cells
Ikebukuro et al., 2006	Mouse	Tolerance induction in allogeneic pancreatic islets
Kaneda et al., 2005	Rat	Induction of tolerance for lung transplantation
Takada et al., 2006	Mouse	Prevention of senile osteoporosis
Taira et al., 2005	Rat	Prevention of type 1 diabetes
Nakamura et al., 2004	Mouse	Prevention of GVHD
Esumi et al., 2003	Rat	Induction of tolerance for allogeneic leg transplantation
Ichioka et al., 2002	Mouse	Prevention of senile osteoporosis

BM cells mainly include HSCs and MSCs. MSCs are essential for supporting hematopoiesis in the BM. HSCs can normally proliferate in major histocompatibility complex (MHC)-compatible MSCs even in allogeneic microenvironments. Because the BMCs are directly injected into bone, IBM-BMT circumvents the risk of MSCs being trapped in the lung and liver. And because both MSCs and HSCs are transplanted, hematopoiesis can be rapidly restored. Moreover, IBM-BMT can prevent the risk of graft rejection, even with the use of a mild conditioning regimen (Kushida et al., 2001).

## IBM-BMT for treatment of RA

RA is an AD that results in a chronic, systemic inflammatory disorder that may affect many tissues and organs. RA primarily affects joints, but it also affects other organs such heart, kidney, and blood vessels (Turesson et al., 2003). Its pathophysiology indicates that TNFα drives synovial inflammation and joint destruction. The synovial cells include both fibroblast-like and macrophage-like synoviocytes. Fibroblast-like synoviocytes show abnormal behavior in RA (Scott et al., 2010). About 50% of RA is caused by genetic abnormalities (van der Woude et al., 2009). The classification criteria for RA by the American College of Rheumatology (2010), and the treatment options, are summarized in the work by Scott et al (Scott et al., 2010). Here we talk about stem cell therapy for the treatment of RA in basic experiments and clinical applications.

SKG/Jcl mice are a murine model for RA. BM cells of C57BL/6J mice were transplanted into SKG/Jcl mice using IBM-BMT, and the hematolymphoid cells in the recipient mice were reconstituted by donor-derived cells. There was no evidence of arthritis in the SKG/Jcl mice at 12 months after transplantation. Moreover, IBM-BMT has been shown to normalize the percentages of Treg (Foxp3+/CD4+) cells, the percentages of receptor activator of NF-kB ligand+ cells on the CD4+ T cells and the serum levels of TNFα, IL-1, and IL-6. One report demonstrated that IBM-BMT is a viable method of immunological manipulation that suppresses the severe joint destruction and bone absorption in SKG/Jcl mice and lends further credence to the use of this methodology in humans with intractable RA (Kushida et al., 2009). Human UC-derived MSCs have been discussed as a possible treatment for RA in the clinical setting. TNFα and IL-6 decreased and CD4+ CD25+ Foxp3+ T cells increased, in active RA patients after UC-derived MSCs were infused, and the UC-derived MSCs survived for 3–6 months, suggesting that treatment with MSCs would benefit RA patients (Wang et al., 2013). Expression of IL-17, IL-6, and TNFα were inhibited when allogeneic UC-derived MSCs were cultured with peripheral blood mononuclear cells (PBMCs) from RA patients, suggesting that MSCs can prevent the expression of these cytokines and that they have therapeutic potential in the treatment of RA (Wang et al., 2012).

## Malignant tumors treated with IBM-BMT + thymus transplantation (TT)

Donor lymphocyte infusion (DLI) is a useful method for the treatment of malignant tumors, but it also induces GVHD. However, IBM-BMT has been shown to prevent not only graft failure but also GVHD in animals, even when the radiation dose is reduced (Nakamura et al., 2004). Thus, IBM-BMT plus DLI were used to treat malignant tumors (fibrosarcomas) induced by a tumor cell line (methA). DLI (CD4^−^ spleen cells) can prevent GVHD, but the tumor growth was not suppressed, indicating that CD4+ cells play important roles in graft-versus-tumor (GVT) and GVHD. Our previous results showed that IBM-BMT plus DLI (CD4^−^lymphocytes) suppressed not only GVHD but also tumor growth (Suzuki et al., 2005). Moreover, the combination of DC, IBM-BMT and DLI showed even better results than the combination of IBM-BMT and DLI in the treatment of solid tumors (Mukaide et al., 2007).

The thymus regulates the production, proliferation and functions of T cells. BMT + TT has been shown to be useful in the treatment of ADs in the MRL/Lpr mouse, because the allogeneic T cells newly-developed by TT are naïve T cells, which show less Fas expression and more resistance to apoptosis than the activated memory T cells with their high Fas expression. We found that the combination of allogeneic IBM-BMT + adult TT from the same donor is effective in mice with solid tumors, as it can induce high thymopoiesis, preserving strong GVT effects without inducing a severe graft-versus-host reaction (GVHR). Meth A sarcoma cells were subcutaneously inoculated into mice, and IBM-BMT + adult TT was then used to treat these mice when the tumor had grown to 5 mm. In tumor-bearing mice, tumor growth was more strongly inhibited by IBM-BMT + adult TT than by IBM-BMT alone. The numbers of CD8^+^ T cells that infiltrated the tumors, and the number of apoptotic tumor cells, both significantly increased in the mice treated with IBM-BMT + adult TT. IBM-BMT + adult TT prevented tumor development with mild GVHR resulting from the induction of high thymopoiesis and a strong GVT effect in the tumor-bearing mice. The number of CD4^+^ FoxP3^+^ cells was lower in the mice treated with IBM-BMT + adult TT than in those treated with IBM-BMT alone. Furthermore, the numbers of CD8^+^ cells infiltrating the tumor and the levels of IFN-γ were higher in the mice treated with IBM-BMT + adult TT than in those treated with IBM-BMT alone (Miyake et al., 2009). Although T regs have been reported to suppress the GVHR induced by CD4^+^T cells, they did not reduce the GVT induced by CD8^+^ T cells (Edinger et al., 2003). Tumors were suppressed to a greater extent as a result of the increased CD4^+^ and CD8^+^ T cells and decreased number of Gr-1^+^/CD11b^+^ myeloid suppressor cells and Foxp3^+^/CD4^+^ T regs, Moreover, the production of CD62L^−^CD44^+^ effector memory T cells and IFN-γ were also higher (Zhang et al., 2011).

IBM-BMT seems to be better than co-transplantation of HSCs and cultured MSCs, mainly because the number of functional MSCs may drop after being cultured *in vitro*, and cultured MSCs also are trapped by the liver and lung in the case of IV-BMT. Umbilical cord blood (UCB) can also be used a source of stem cells for transplantation, although the numbers are generally insufficient to allow this to be used as a general source. IBM thus appears to be the best choice for allogeneic transplantation, despite the limited number of stem cells that can be directly transplanted into the bone cavity. In conclusion, IBM-BMT can efficiently transplant both HSCs and MSCs, is useful to treat intractable diseases such as RA and malignant tumors, and in the future may be useful for treating various intractable diseases.

### Conflict of interest statement

The authors declare that the research was conducted in the absence of any commercial or financial relationships that could be construed as a potential conflict of interest.
